# Focused Review: Potential Rare and Atypical Symptoms as Indicator for Targeted COVID-19 Screening

**DOI:** 10.3390/medicina57020189

**Published:** 2021-02-23

**Authors:** Swee Li Ng, Yong Sze Ong, Kooi Yeong Khaw, Siew Phooi Teh, Ching Siang Tan, Long Chiau Ming, Kok-Gan Chan, Learn-Han Lee, Bey-Hing Goh

**Affiliations:** 1 Biofunctional Molecule Exploratory Research Group (BMEX), School of Pharmacy, Monash University Malaysia, Bandar Sunway 47500, Malaysia; sally.ngsl06@gmail.com (S.L.N.); Ong.YongSze@monash.edu (Y.S.O.); Khaw.KooiYeong@monash.edu (K.Y.K.); sphooi84@gmail.com (S.P.T.); 2 Health and Well-Being Cluster, Global Asia in the 21st Century (GA21) Platform, Monash University Malaysia, Bandar Sunway 47500, Malaysia; 3 Tropical Medicine & Biology Platform, Monash University Malaysia, Bandar Sunway 47500, Malaysia; 4 College of Pharmaceutical Sciences, Zhejiang University, Hangzhou 310027, China; 5 School of Pharmacy, KPJ Healthcare University College, Nilai 71800, Malaysia; 6 PAP Rashidah Sa’adatul Bolkiah Institute of Health Sciences, Universiti Brunei Darussalam, Gadong BE1410, Brunei; longchiauming@gmail.com; 7 Division of Genetics and Molecular Biology, Institute of Biological Sciences, Faculty of Science, University of Malaya, Kuala Lumpur 50603, Malaysia; kokgan@um.edu.my; 8 International Genome Centre, Jiangsu University, Zhenjiang 212013, China; 9 Novel Bacteria and Drug Discovery Research Group, Microbiome and Bioresource Research Strength, Jeffrey Cheah School of Medicine and Health Sciences, Monash University Malaysia, Subang Jaya 47500, Malaysia

**Keywords:** COVID-19, symptoms, screening, detection, infection

## Abstract

The global pandemic of the coronavirus disease 2019 is a known consequence of infection of severe respiratory syndrome coronavirus-2 (SARS-CoV-2). It has affected nations worldwide with soaring number of cases daily. Symptoms such as fever, cough, and shortness of breath, diarrhea, nausea and vomiting are commonly presented in COVID-19 patients. This focused review aims to discuss these uncommon and atypical COVID-19 symptoms that may be presented which might affect neurological, cardiovascular, cutaneous and ocular systems and their possible mode of actions. Nonetheless, there are some cases of reported uncommon or atypical symptoms which may warrant healthcare professionals to be aware of, especially when in contact with patients. The knowledge and information concerning these symptoms might be able to provide additional cues for healthcare professional by subjecting patients to COVID-19 screening. Meanwhile, it might be able to further enhance the alertness and additional precautions being taken by healthcare personnel, which eventually lead to reduced risk of infections.

## 1. Introduction

The pandemic of the coronavirus disease 2019 (COVID-19) has affected the global population since the first detection in late 2019. To date, it has recorded over 86 million confirmed cases and attained a mortality rate of about 4% (as of 9 January 2021) [1,2]. Patients with overt cardiovascular disease are particularly susceptible to COVID-19 and may increase the mortality rate [3].

The culprit of this disease, severe respiratory syndrome coronavirus-2 (SARS-CoV-2) belongs to the *Coronaviridae* family, *Betacoronavirus* genus and Orthocoronaviridae subfamily. The spike glycoprotein at the N-terminal region of SARS-CoV-2 acts as a receptor binding domain which binds to the human angiotensin converting enzyme 2 (ACE2) of the host cell. This leads to fusion between viral and host cell membranes to allow entry of coronavirus into target host cells [4]. Upon being infected with SARS-CoV-2, patients may present without symptoms or with symptoms, ranging from mild, moderate, severe to critical, with clinical classifications outlined by the Chinese National Health Committee [5]. Symptoms commonly presented in COVID-19 patients include fever, fatigue respiratory symptoms like cough and shortness of breath, and gastrointestinal symptoms like diarrhea, nausea and vomiting [6]. Although there is a list of symptoms presented in the COVID-19 infected patients, they are quite identical to other normally occurring illnesses such as flu or diarrhoea. This has further made the task of identifying and quarantining infected patients a real great challenge.

Interestingly, there are an increasing number of cases that reported a variety of other symptoms that may not be commonly manifested in COVID-19, which might provide some critical useful hints for healthcare personnel to pinpoint those who might be infected and provide the necessary actions accordingly. Moreover, the changes in hematological parameters in SARS–CoV–2-infected patients are imperative to understand the pathophysiology of the disease and useful information as early clues to diagnosis [7]. Since the very first reported case of COVID-19 in 2019, researchers have been working relentlessly in order to design the effective drugs or specific vaccine for this disease; however, the discovery process has faced various great challenges. In actual fact, the World Health Organization (WHO) released a statement very recently stating that there is no silver bullet for this pandemic, which has reflected greatly on the gravity of the situation [8].

As of 9 January 2021, different countries have approved and started COVID-19 vaccinations among their populations using vaccines from Pfizer, Moderna and AstraZeneca, with large numbers of vaccines at phase three of clinical trials [9,10,11]. Other management of COVID-19 includes the use of antivirals and antimalarial drugs, immune-based therapy and supportive oxygen therapy for those who are in severe condition [12]. Previous measures in controlling the pandemic continue to be practiced in this early phase of vaccinations. These include (1) social distancing, regular hand washing, wearing face masks in public and (2) testing, isolating and treating patients besides tracing and quarantining close contacts done by the healthcare facilities. Thus, it is of the utmost importance for healthcare providers to recognize and detect all possible symptoms in COVID-19 when treating patients to allow testing, confirmation and isolation to be done in a timely manner to prevent an outbreak and protect everyone.

This review focuses on the identification and description of rare and atypical symptoms that may aid targeted screening. Therefore, this article has pinpointed the less common and atypical symptoms that may be presented in infected patients, with its possible mechanism of action, which may shed some light for us in enhancing the effectiveness in identification and targeted screening processes (Table 1).

## 2. Research Methodology

The primary focus was to search for all relevant literature published on the rare and atypical symptoms of COVID-19. A systematic search was performed using Google Scholar until 9 January 2021. Based on the information from the World Health Organization [9,27], the key words used in our search strategy include “coronavirus disease”, “coronavirus”, “COVID-19”, “SARS-CoV-2” combined with “symptoms”, “symptom”, “manifestations”, “manifestation”, “clinical”, “characteristics”, “signs”, “rare”, “atypical”, “uncommon”, “neurological”, “ocular”, “eye”, “optical”, “cardiovascular”, “cutaneous”, “skin”, “dermatological”, using Boolean operators. Search results in English either as original language or translated were included. As it was known that typical symptoms of COVID-19 were fever, cough, respiratory and gastrointestinal symptoms, literatures focusing on these topics were excluded from this review. The reference list from relevant articles was also screened to identify additional literatures. In addition, the reference lists were supplemented by manual searching.

## 3. Neurological Symptoms

SARS-CoV-2 is known to target angiotensin converting enzyme-2 (ACE2), which are expressed in various human organs and tissues including the nervous system, skeletal muscles and mucosa of oral cavity [28]. Therefore, neurological symptoms may be exhibited in COVID-19 patients as the presence of ACE2 in nervous symptoms. Therefore, it is not surprising to observe the existence of hyperemic brain, oedema and neurons degeneration in autopsies of COVID-19 patients [5]. Based on those eventual findings, we could predict a list of neurological symptoms that could be manifested via the central nervous system, peripheral nervous system and skeletal muscular system if they have been affected or injured. The postulations include dizziness, headache, changes in mental status, acute cerebrovascular disease, ataxia, and seizures are amongst the symptoms involving central nervous symptoms. Meanwhile, peripheral nervous symptoms such as anosmia, ageusia, impaired vision and nerve pain could be observed as well. In fact, these symptoms are exhibited during the early phase of the infection with a median of 1–2 days; thus, clinicians and neurologists are encouraged to seek out these symptoms to rule out the possibility of COVID-19 if there is no prehistorical record of neuronal condition [29]. The observations have highlighted patients with a severe condition who exhibited a lowered lymphocyte count and increased D-dimer level are more likely to experience neurological symptoms, especially centrally involving the central nervous system [17,29,30]. This unique correlation may provide an additional hint or idea for medical professionals in establishing a further diagnosis on these suspected patients.

The effect of COVID-19 on ACE2 which are found on endothelial cells may play a role in proinflammatory and vasoconstriction of endothelial and leading to stroke and other end-organ damage. This could be accountable for the stroke that is being identified as one of the most common neurological symptoms in COVID-19 patients. Thus far, it has been manifested in almost half of the total subjects, with 77% of them having experienced the large-vessel occlusion [17]. The increased levels of D-dimer, intravascular platelet activation, fibrinogen, prothrombin time and activated partial thromboplastin time that were seen in these patients may explain the hypercoagulation events in COVID-19. Lupus anticoagulants, anticardiolipin, anti-β2-glycoprotein I antibodies and antiphospholipid antibodies were found in patients with neurological symptoms leading to thrombotic events [31,32]. This may warrant for anticoagulants and/or antiplatelets for treatment and prevention of thrombotic events in COVID-19. However, further trials are needed to study their efficacies.

In sum, a list of neurological symptoms and postulations that may occur based on existing autopsy findings and the effect of SARS-CoV-2 on the ACE2 enzyme were presented above. The correlation of a lowered lymphocyte count and increased D-Dimer to severe disease and central neurological symptoms is apparent. Nevertheless, it is still unclear if this is associated with merely severe disease rather than the development of neurological symptoms.

Another rare neuromuscular symptom displayed among COVID-19 patients is Guillain-Barre syndrome (GBS). The common onset of this symptom was a few days after flu-like symptoms; however, in some cases, GBS was presented first [17]. The mechanism of GBS in COVID-19 is still unknown, however it is known that COVID-19 infection initiates immune response and inflammation, whereas GBS is categorized as an auto-immune disorder. It is still unclear whether COVID-19 infection has induced the production of an antiganglioside antibody that is implicated in GBS [33]. On the other hand, the formation of cytokine storm condition in COVID-19 may be implicated in the damage of neurons and lead to the manifestation of the various neuromuscular symptoms. Among the commonly manifested muscle symptoms, myalgia is one of the most commonly found in COVID-19 patients. Intensive studies done have also found that these patients were found to have high levels of creatine kinase, neutrophils, C-reactive protein, D-dimer and lactate dehydrogenase [29,34]. Comparatively, adult patients may be more prone to myalgia condition in addition to multi-organ damage, renal and liver abnormalities.

Taste and olfactory impairment are also exhibited in mild¬ to moderate ambulatory patients during the early phase of the disease, which will resolve together with other COVID-19 symptoms within a few weeks [35,36,37]. Patients may experience dysfunction in both taste and olfactory systems or either one dysfunction of these systems [38,39,40]. In few cases, impairment in taste and/or smell were reported as the only symptoms experienced without the typical symptoms, i.e., cough, shortness of breath, fever [41,42,43]. Previously, it was unknown whether olfactory dysfunction in COVID-19 was due to nasal congestion, rhinorrhea, olfactory nerves impaired by SARS-CoV-2 or other co-morbidities. It is speculated that acute anosmia might happen when CD68^+^ macrophages carry SARS-CoV-2 to target olfactory epithelium, which can regenerate and repair quickly post viral infection. In both self-reported surveys and quantitative studies, a notable number of positive COVID-19 patients had olfactory and/or gustatory impairment, and these patients did not exhibit clinically significant nasal congestion, olfactory cleft obstruction, sinusitis and rhinorrhea symptoms [39,44,45]. There is an exception, which the study by Klopfenstein et al. demonstrated, that more than half of the patients had anosmia and rhinorrhea [46]. The incidence of gustatory and olfactory impairment seems to be higher in European patients than Asian patients, which could be due to genetic mutation of the virus strain, under-reporting of these cases or a lack of assessment of these symptoms in an Asian setting [41,46]. A further analysis on prevalence of anosmia across gender has shown a no difference according to a systematic review by Chung et al., although a few studies have shown that anosmia is more common in female and younger populations [41,47].

Other neurological symptoms include headache, reduced consciousness, confusion, dizziness and seizures, which have been reported in COVID-19 patients. However, these symptoms are non-specific, less prevalent than respiratory symptoms and tend to be over-looked. Lu et al. reported that no acute symptomatic seizures and status epilepticus were reported, as the virus and risk factors are considered as insignificant risks [48]. However, the available data for epilepsy are still limited to conclude its association with COVID-19. Alternatively, these symptoms could be considered as an encephalopathic state but cannot be categorized confidently due to a lack of magnetic resonance imaging scan and invasive procedures to confirm the diagnosis. Further observations have further pinpointed the encephalopathy condition due to encephalitis; especially meningoencephalitis is commonly diagnosed in patients with SARS-CoV-2 infection. It could be explained mechanistically that SARS-CoV-2 may enter the central nervous system through hematogenous entry assisted by ACE2 protein, retrograde synaptic transmission via olfactory nerve or increased permeability of blood–brain barrier and blood–cerebrospinal fluid barrier as a result of systemic hyperinflammation. However, in most cases, except two cases of meningoencephalitis, the cerebrospinal fluid was not found to contain the virus [49,50,51]. This is possibly due to the immune-mediated nature of COVID-19, encephalopathy due to endothelial injury and thrombotic microangiopathy-like state or suboptimal sensitivity of polymerase chain reaction (PCR) for SARS-CoV-2 instead of crossing the blood–brain barrier [17].

## 4. Cutaneous Symptoms

Cutaneous features associated with COVID-19 can be categorized by two categories: inflammatory/exanthematous eruptions and vasculopathic/vasculitic lesions [52]. Inflammatory/exanthematous eruptions include urticarial lesions, erythema multiforme-like/maculopapular/morbilliform rash and papulovesicular exanthem. Pseudo-chilblain, acro-ischaemia, livedo reticularis, distal necrosis and purpuric/petechiae vasculitis rash fall under the vasculopathic/vasculitic lesions category. Currently, established reviews and systematic reviews that have been found in literature have generally showed that skin lesions are highly varied and may resolve unprompted. A meta-analysis reported that maculopapular rash was the most prevalent skin manifestation with latency of at least 8 days amongst 9.9% of COVID-19-positive patients [18]. Two other notable skin lesions are pseudo-chilblain and erythema rashes, which localized mainly at the trunk and extremities of patients. Pruritus is commonly associated with both lesions, followed by pain and burning sensation [53,54,55]. Acro-ischaemia found in severe COVID-19 patients were hypothesized due to hypercoagulation, whereas pseudo-chilblain in asymptomatic and mild young patients were due to coagulation disorder or hypersensitivity [52,56]. Erythema rashes prevalent in middle-aged adults were suggested to be caused by virus-specific T-cells. The onset of the skin lesions is varied, but under 30 days, can occur as the first symptom or after onset of non-cutaneous COVID-19 symptoms. The mean duration of skin lesions manifestation is around 9 days though the observations done. Matar and colleagues showed that the severity and mortality rates for patients with rashes were significantly higher than chilblains [54]. Other atypical skin manifestations that have been reported which require further investigation include atypical erythema nodosum, atypical Sweet syndrome, Kawasaki disease-like presentation and polymorphic patterns [19].

## 5. Ocular Symptoms

Although evidence is limited, there are cases of COVID-19 patients who have manifested ocular symptoms. Further studies done by researchers have detailed the proposed mechanism of infection. It is proposed that conjunctiva is the inoculation site of the SARS-CoV-2 from infected droplets. Along with that, viral migration may occur at the upper respiratory tract through the nasolacrimal duct or hematogenous, together with the involvement of the lacrimal gland [57]. This is mainly due to the fact of the presence of renin-angiotensin system or ACE2 receptor in the aqueous humour of the human eye [58]. However, more evidence is required to support ocular infection of SARS-CoV-2 through ACE2. The overall rate of ocular manifestations among COVID-19 patients is established at a range from 1% to 32% [58,59]. Ocular symptoms are varied, some cases are reported as first presentation, whereas others reported as secondary to COVID-19 progression. The most common ophthalmologic symptom reported is conjunctivitis with about 0.7% patients reported as first symptom [58,60,61]. Other symptoms reported include chemosis, epiphora, conjunctival hyperaemia, keratoconjunctivitis, haemorrhagic conjunctivitis with pseudomembranous and ophthalmoparesis [62,63,64]. Ocular symptoms have been associated with some cases of severe COVID-19, in which patients have higher white blood cells, neutrophils, procalcitonin, C-reactive protein and lactate dehydrogenase comparing to patients without ocular symptoms [59,65]. However, another meta-analysis reported that ocular symptoms were not associated with severe disease [66]. Inconsistency of PCR positive results for SARS-CoV-2 in tear/conjunctival were observed. Some cases have reported that the virus can be detected in tears and conjunctival secretion sampled from COVID-19 patients with conjunctivitis [67,68]. A number of cases have demonstrated that some patients were positive for the virus in tear even without experiencing conjunctivitis, whereas other patients had conjunctivitis with negative PCR tests [69]. Only about 2% to 3.5% of ocular samples retrieved from COVID-19-positive patients were tested positive for the virus [21,22,58]. Thus, the relation between positive SARS-CoV-2 in tear/conjunctival swab and ocular symptoms including conjunctivitis is still uncertain.

## 6. Cardiovascular Symptoms

Although most evidence are mainly anecdotal with a lack of systematic review, cardiovascular manifestations do exist in some COVID-19 patients regardless of any prior cardiovascular diagnosis. Suggested theories of cardiovascular involvement in COVID-19 include direct myocardial injury, cytokine storm, pre-existing cardiovascular disease co-morbidities and the use of ACE inhibitors and angiotensin receptor blockers (limited evidence) [70]. Myocardial injury caused by myocardial ischemia and myocarditis were showed as elevation in cardiac troponin-I levels and other inflammatory markers including ferritin, C-reactive protein, interleukin-6, interferon-γ, tumor necrosing factor-α and lactate dehydrogenase [71,72]. Patients with elevated cardiac markers had higher prevalence of pre-existing cardiovascular disease and were more likely to be admitted into intensive care unit along with the ventilation support. They normally ended up with poor prognosis and high mortality. Myocardial injury can also result from acute respiratory distress syndrome due to oxidative stress and potentially inflammation-induced myocardial apoptosis [70]. Acute myocarditis and ventricular arrythmia may be first presented in COVID-19 with arrhythmia which might occurred due to electrolyte and haemodynamic imbalances. The increase of troponin level would directly correlate with malignant ventricular arrhythmia [73]. Thus, continuous electrocardiogram monitoring is highly recommended when drugs that prolong QT intervals like hydroxychloroquine or chloroquine are given as COVID-19 treatment [6]. In addition, venous thromboembolism (VTE) is also observed in COVID-19 patients due to inflammatory states, old age, comorbidities, respiratory failure, immobility, low lymphocyte count and high D-dimer count. High levels of D-dimer and fibrin degradation products have been associated with severe infection and mortality [73,74]. The incidence of VTE in severe COVID-19 was 25% out of 81 patients, of which 8 of them died [75]. Patients with impaired left ventricular and right ventricular function as well as tricuspid regurgitation > grade 1 were significantly associated with higher mortality [72]. It is well recognized that development of heart failure is also significantly more prevalent in non-survivors compared to survivors [76]. Eventually, cardiovascular manifestations in COVID-19 may overlap and mask with the respiratory symptoms, thus cardiovascular involvement should not be overlooked when treating COVID-19 patients.

## 7. Conclusions

People infected with COVID-19 have commonly reported with respiratory symptoms including fever, cough and shortness of breath, besides gastrointestinal symptoms including diarrhea, nausea and vomiting. As we learn more about COVID-19, there are more varieties of symptoms manifested in COVID-19 as discussed above, involving the neurological, cutaneous, cardiovascular and ophthalmological system. Thus, it is highly vital that all healthcare professionals working and involved in direct contact with patients be vigilant of the variety of possible COVID-19 symptoms and consider testing the patients for COVID-19 during this pandemic.

## Figures and Tables

**Table 1:** Prevalence of atypical and rare symptoms of COVID-19.

Symptoms	Comments	Prevalence	Citations
Anosmia	Prevalent in younger patients, females, occurs in early stages of COVID-19	35.8%	Favas et al. [13]
		35.7–85.6%	Wang et al. [14]
		35%	Abdullahi et al. [15]
Ageusia		38.5%	Favas et al. [13]
		33.3–88.8%	Wang et al. [14]
		33%	Abdullahi et al. [15]
Headache	Tend to be found in young patients, occurs in early stages of COVID-19	14.7%	Favas et al. [13]
		12%	Abdullahi et al. [15]
		10.9%	Pinzon et al. [16]
Acute cerebrovascular disease	Onset: 9–10 days; Prevalent in older patients; associated with severe form of COVID-19	2.3%	Favas et al. [13]
		3%	Abdullahi et al. [15]
		4.4%	Pinzon et al. [16]
Ischaemic stroke		2.1%	Favas et al. [13]
Haemorrhagic stroke		0.4%	Favas et al. [13]
Guillain–Barré syndrome	Onset: 3–24 days	4 case reports	Wang et al. [14]
		73.9% (*n* = 17)	Ghannam et al. [17]
Maculopapular rash	Onset: 8 days, commonly found in females	37.5%	Rocha et al. [18]
		38%	Conforti et al. [19]
Chilblain-like	In younger patients with median age: 20; median onset: 5 days; associated with mild disease	10%	Rocha et al. [18]
		12.8%	Conforti et al. [19]
		18%	Lee et al. [20]
Acro-ischemia	Associated with severe disease; Onset: 19 days	9%	Lee et al. [20]
Kawasaki disease-like presentation	Onset: 3.5 days; commonly found in males	6.9%	Lee et al. [20]
		0.8%	Conforti et al. [19]
Polymorphic patterns	Rare symptoms; requires further investigation	1.4%	Conforti et al. [19]
Generalized pruritus		1.2%	Conforti et al. [19]
Atypical erythema nodosum		0.5%	Conforti et al. [19]
Atypical Sweet syndrome		0.2%	Conforti et al. [19]
Ocular redness	Can be presented as first symptom or developed during disease progression	10.9%	Aggarwal et al. [21]
Conjunctivitis		7%	Aggarwal et al. [21]
		8.3%	Cao et al. [22]
Conjunctival chemosis		4.4%	Aggarwal et al. [21]
Acute myocardial injury	Associated with severe form of COVID-19; Predominant in males, median age: 56	15%	Vakhshoori et al. [23]
		18%	Gong and Guo [24]
		15%	Potore et al. [25]
Venous Thromboembolism	More common in COVID-19 patients in intensive care unit, increased odds of mortality	15%21%	Potore et al. [25] Malas et al. [26]
